# The mitochondrial unfolded protein response is activated upon hematopoietic stem cell exit from quiescence

**DOI:** 10.1111/acel.12756

**Published:** 2018-03-24

**Authors:** Mary Mohrin, Andrew Widjaja, Yufei Liu, Hanzhi Luo, Danica Chen

**Affiliations:** ^1^ Program in Metabolic Biology, Nutritional Sciences & Toxicology University of California Berkeley CA USA; ^2^ Present address: Calico Life Sciences South San Francisco California USA

**Keywords:** aging, hematopoietic stem cells, mitochondria, mitochondrial unfolded protein response, SIRT7, stem cell quiescence, stem cells

## Abstract

The mitochondrial unfolded protein response (UPR ^mt^), a cellular protective program that ensures proteostasis in the mitochondria, has recently emerged as a regulatory mechanism for adult stem cell maintenance that is conserved across tissues. Despite the emerging genetic evidence implicating the UPR ^mt^ in stem cell maintenance, the underlying molecular mechanism is unknown. While it has been speculated that the UPR ^mt^ is activated upon stem cell transition from quiescence to proliferation, the direct evidence is lacking. In this study, we devised three experimental approaches that enable us to monitor quiescent and proliferating hematopoietic stem cells (HSCs) and provided the direct evidence that the UPR ^mt^ is activated upon HSC transition from quiescence to proliferation, and more broadly, mitochondrial integrity is actively monitored at the restriction point to ensure metabolic fitness before stem cells are committed to proliferation.

## 1 INTRODUCTION

Adult stem cells persist throughout the entire lifespan of an organism to repair tissue damage and maintain tissue homeostasis. Among their evolved adaptations are elaborate cellular protective programs that ensure stem cell integrity, tissue homeostasis, and organismal survival (Biteau, Hochmuth & Jasper, 2008; Brown et al., 2013; Ito et al., 2004; Rando, 2006; Renault et al., 2009; Rossi, Jamieson & Weissman, 2008; Rossi et al., 2007; Sahin & Depinho, 2010; Sperka, Wang & Rudolph, 2012; Walter et al., 2015). The mitochondrial unfolded protein response (UPR^mt^), a cellular protective program that ensures proteostasis in the mitochondria, has recently emerged as a regulatory mechanism for adult stem cell maintenance that is conserved across tissues (Berger et al., 2016; Mohrin et al., 2015; Zhang et al., 2016). This protective program is dysregulated during physiological aging, contributing to the functional deterioration of stem cells, tissue degeneration, and shortened organismal lifespan (Mohrin et al., 2015; Zhang et al., 2016). In addition to the UPR^mt^, deregulation of compensatory mitochondrial protective programs such as mitophagy and mitochondrial dynamics leads to compromised stem cells, further underscoring the importance of mitochondrial integrity in stem cell maintenance (Ho et al., 2017; Ito et al., 2016; Luchsinger, de Almeida, Corrigan, Mumau & Snoeck, 2016; Vannini et al., 2016).

Despite the emerging genetic evidence implicating the UPR^mt^ in stem cell maintenance, the underlying molecular mechanism is unknown. The UPR^mt^ is a nascent cellular pathway that is activated when cells experience mitochondrial protein folding stress and retrograde signaling from the mitochondria to the nucleus triggers transcriptional activation of nuclear‐encoded mitochondrial chaperones and proteases as well as repression of translation to reestablish proteostasis (Haynes, Fiorese & Lin, 2013; Haynes & Ron, 2010; Mohrin et al., 2015; Munch & Harper, 2016; Zhao et al., 2002). Primarily characterized in *C. elegans*, the UPR^mt^ is activated during a developmental stage when there is a burst of mitochondrial biogenesis (Houtkooper et al., 2013; Lin et al., 2016; Merkwirth et al., 2016; Nargund, Pellegrino, Fiorese, Baker & Haynes, 2012; Pellegrino et al., 2014; Tian et al., 2016). It is therefore speculated that in stem cells, the UPR^mt^ is activated under a physiological condition when mitochondrial biogenesis is induced. Adult stem cells frequently exit the cell cycle and are predominantly found in the quiescent (G0) state, where the number of mitochondria is low and glycolysis is the primary metabolic pathway to support energy production (Folmes, Dzeja, Nelson & Terzic, 2012; Takubo et al., 2013; Warr & Passegue, 2013; Yu et al., 2013). As stem cells transit from quiescence to proliferation, mitochondrial biogenesis is induced to enable metabolic reprogramming from glycolysis to oxidative phosphorylation to meet increasing energy demands. Because a major event during the transition from quiescence to proliferation is mitochondrial biogenesis, it raises the possibility that the UPR^mt^ is activated during this transition. However, the direct evidence is lacking. In this study, we devised three experimental approaches that enable us to monitor quiescent and proliferating stem cells and directly test this hypothesis.

**Figure 1:**
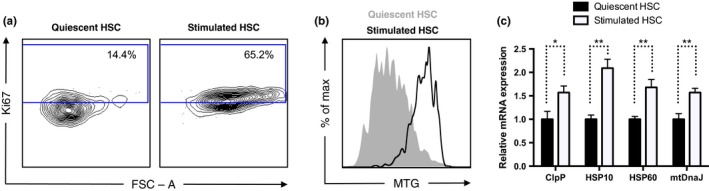
The UPR ^mt^ is activated upon HSC transition from quiescence to proliferation ex vivo. (a) Cell cycle analysis of HSCs using Ki‐67 staining showing increased proliferation in HSCs stimulated by ex vivo culture with cytokines compared to quiescent HSCs freshly isolated from mouse bone marrow. (b) MitoTracker Green staining showing increased mitochondrial mass in HSCs stimulated to proliferate via ex vivo culture with cytokines compared to quiescent HSCs freshly isolated from mouse bone marrow. (c) qPCR showing increased transcription of mitochondrial chaperones and proteases in HSCs stimulated to proliferate via ex vivo culture with cytokines compared to quiescent HSCs freshly isolated from mouse bone marrow. *n* = 3

We tested this hypothesis in hematopoietic stem cells (HSCs), immunophenotypically defined as Lin^−^c‐Kit^+^Sca1^+^CD150^+^CD48^−^. About 90% of HSCs reside in a quiescent state under homeostatic conditions (Pietras, Warr & Passegue, 2011). We isolated HSCs from mouse bone marrow and stimulated them to exit quiescence ex vivo upon culture with cytokines. We first confirmed that HSCs stimulated with cytokines were actively proliferating (Figure 1a) and that mitochondrial mass was increased in HSCs upon proliferation (Figure 1b). Compared to freshly isolated quiescent HSCs, proliferating HSCs stimulated with cytokines exhibited increased expression of mitochondrial chaperones and proteases at the transcriptional level (Figure 1c). Because mitochondrial biogenesis upon HSC transition from quiescence to proliferation is regulated at the translational level mediated by mTOR (Chen et al., 2008; Gan et al., 2010; Gurumurthy et al., 2010; Morita et al., 2013; Nakada, Saunders & Morrison, 2010), increased expression of mitochondrial chaperones and proteases at the transcriptional level reflects de novo activation of the UPR^mt^.

**Figure 2:**
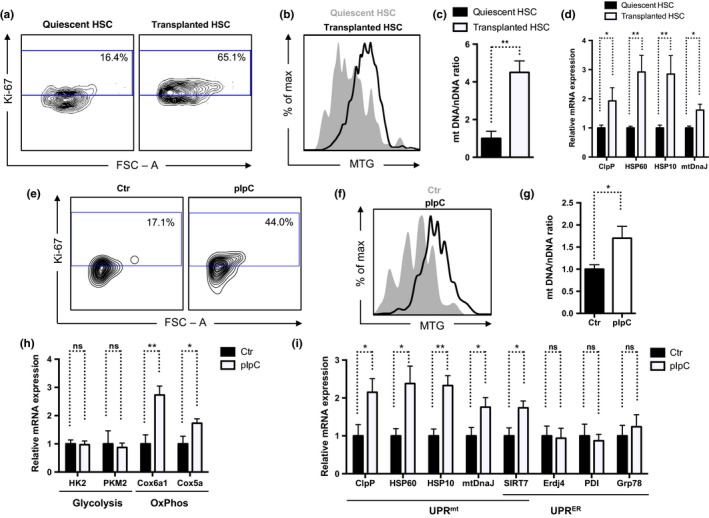
The UPR ^mt^ is activated upon HSC transition from quiescence to proliferation in vivo. (a) Cell cycle analysis of HSCs using Ki‐67 staining showing increased proliferation in HSCs stimulated by in vivo transplantation (2 week post‐transplantation) compared to quiescent HSCs freshly isolated from mouse bone marrow. (b, c) MitoTracker Green staining (b) and quantification of the mitochondrial to nuclear DNA ratio (c) showing increased mitochondrial mass in HSCs stimulated to proliferate via in vivo transplantation compared to quiescent HSCs freshly isolated from mouse bone marrow. *n* = 3. (d) qPCR showing increased transcription of mitochondrial chaperones and proteases in HSCs stimulated to proliferate via in vivo transplantation compared to quiescent HSCs freshly isolated from mouse bone marrow. *n* = 3. (e) Cell cycle analysis of HSCs using Ki‐67 staining showing increased proliferation in HSCs stimulated by pIpC treatment (24 hr after treatment) compared to quiescent HSCs isolated from untreated mouse bone marrow. (f, g) MitoTracker Green staining (f) and quantification of the mitochondrial to nuclear DNA ratio (g) showing increased mitochondrial mass in HSCs stimulated to proliferate via pIpC compared to quiescent HSCs isolated from untreated mouse bone marrow. *n* = 3. (h, i) qPCR showing increased transcription of oxidative phosphorylation genes and mitochondrial chaperones and proteases, but not glycolysis genes and ER stress response genes in HSCs stimulated to proliferate via pIpC treatment compared to quiescent HSCs isolated from untreated mouse bone marrow. *n* = 3

We further validated these results by stimulating HSCs to exit quiescence in vivo upon transplantation. Compared to HSCs isolated from untransplanted mice, donor HSCs isolated from transplanted recipient mice 2 weeks post‐transplant were actively proliferating (Figure 2a), had increased mitochondrial mass (Figure 2b,c), and increased expression of mitochondrial chaperones and proteases at the transcriptional level (Figure 2d), indicative of activation of the UPR^mt^.

An alternative approach to model HSC proliferation in vivo is to treat mice with polyinosinic:polycytidylic acid (pIpC), a synthetic double‐stranded RNA (dsRNA) mimetic that stimulates the multiple immune signaling pathways that are activated during a viral infection (Walter et al., 2015). Compared to HSCs isolated from untreated mice, HSCs isolated from mice 24 hr after the pIpC treatment showed increased proliferation (Figure 2e) and mitochondrial mass (Figure 2f,g), and induction of the expression of oxidative phosphorylation genes (Figure 2h) and mitochondrial chaperones and proteases (Figure 2i). In contrast, the expression of glycolysis genes (Figure 2h) and ER stress response genes (Figure 2i) was unchanged. These data are consistent with the notion that proliferative HSCs have increased mitochondrial number, experience a metabolic switch from glycolysis to oxidative phosphorylation, and induce the UPR^mt^ to maintain the mitochondrial homeostasis.

Collectively, these results provide direct evidence that the UPR^mt^ is activated upon HSC transition from quiescence to proliferation (Figures 1 and 2), and more broadly, mitochondrial integrity is actively monitored at the restriction point to ensure metabolic fitness before stem cells are committed to proliferation. Stem cell quiescence is a protective mechanism that prevents cell death and the depletion of the stem cell pool (Cheung & Rando, 2013). Consistent with the activation of the UPR^mt^ at the transition from quiescence to proliferation, dysregulation of the UPR^mt^ results in stem cell death, a reduced stem cell pool, and compromised stem cell self‐renewal (Berger et al., 2016; Mohrin et al., 2015; Zhang et al., 2016). Among the induction of the UPR^mt^ is increased expression of SIRT7 (Figure 2i), which alleviates mitochondrial protein folding stress by repressing NRF1 activity and mitochondrial translation, reduces mitochondrial activity and proliferation, and gives cells more time to recover from stress (Mohrin et al., 2015). Failure to do so leads to HSC death. With aging, SIRT7 becomes inactivated, resulting in increased mitochondrial protein folding stress and functional decline (Mohrin et al., 2015). Identifying molecular regulators of the UPR^mt^ in stem cells opens the door for novel therapeutic opportunities for improving stem cell maintenance, enhancing tissue regeneration, and extending lifespan and healthspan.

## 2 EXPERIMENTAL PROCEDURES

### 2.1 Mice

C57BL/6 mice were housed on a 12:12 hr light:dark cycle at 25°C. All animal procedures were in accordance with the animal care committee at the University of California, Berkeley.

### 2.2 Flow cytometry and cell sorting

Bone marrow cells were obtained by crushing the long bones with sterile PBS without calcium and magnesium supplemented with 2% FBS. Lineage staining contained a cocktail of biotinylated anti‐mouse antibodies to Mac‐1 (CD11b), Gr‐1 (Ly‐6G/C), Ter119 (Ly‐76), CD3, CD4, CD8a (Ly‐2), and B220 (CD45R) (BioLegend). For detection or sorting, we used streptavidin conjugated to APC‐Cy7, c‐Kit‐APC, Sca‐1‐Pacific blue, CD48‐FITC, and CD150‐PE (BioLegend). For congenic strain discrimination, anti‐CD45.1 PerCP and anti‐CD45.2 PE‐Cy7 antibodies (BioLegend) were used. For assessment of cell cycle analysis, Ki‐67 (BioLegend) staining was performed according to the manufacturer's recommendation after cell surface staining. The gates were drawn based on the fluorescence minus one (FMO) control. For mitochondrial mass, bone marrow cells were incubated with 100 nM MitoTracker Green (Invitrogen) for 30 min at 37°C in the dark after cell surface staining. All data were collected on a Fortessa (Becton Dickinson), and data analysis was performed with FlowJo (TreeStar). For cell sorting, lineage depletion or c‐kit enrichment was performed according to the manufacturer's instructions (Miltenyi Biotec). Cells were sorted using a Cytopeia INFLUX Sorter (Becton Dickinson). Antibody details are provided in Table S1.

To stimulate HSCs to exit quiescence, freshly isolated HSCs were cultured ex vivo in IMDM (Invitrogen) supplemented with 5% stem cell FBS (Stem Cell Technologies), 1% penicillin/streptomycin, sodium pyruvate, NEAA, l‐glutamine (Invitrogen), and cytokines (IL‐3 (10 ng/ml), GMCSF (10 ng/ml), SCF (25 ng/ml), IL‐11 (25 ng/ml), Flt3L (25 ng/ml), TPO (25 ng/ml) (PeproTech), and EPO (4 U/ml) (R&D)) for 48 hr. Alternatively, to stimulate HSCs to exit quiescence in vivo, 1 × 10^6^ bone marrow cells were transplanted into lethally irradiated recipient mice. Two weeks post‐transplantation, donor HSCs were isolated via sorting. To induce in vivo* *cycling of HSCs, mice were injected intraperitoneally (i.p.) with 5 mg/kg polyinosinic:polycytidylic acid (Sigma) 24 hr prior to analysis.

### 2.3 mRNA analysis

RNA was isolated from cells using TRIzol reagent (Invitrogen). cDNA was generated using qScript™ cDNA SuperMix (Quanta Biosciences). Gene expression was determined by real‐time PCR using Eva qPCR SuperMix Kit (BioChain Institute) on an ABI StepOnePlus system. All data were normalized to β‐actin expression. PCR primer details are provided in Table S2.

### 2.4 mtDNA/nDNA

The mitochondrial DNA/nuclear DNA (mtDNA/nDNA) ratio was determined by isolating DNA from cells with TRIzol (Invitrogen), as described previously (Lai et al., 2008). The ratio of mtDNA/nDNA was calculated as previously described (Venegas et al., 2011).

### 2.5 Statistical analysis

The number of mice chosen for each experiment is based on the minimum number of mice necessary to have sufficient statistical power and is comparable to published literature for the same assays performed. Mice were randomized to groups, and analysis of mice and tissue samples was performed by investigators blinded to the treatment of the animals. Statistical analysis was performed with Excel (Microsoft). Means between two groups were compared with Student's *t* test. Error bars represent standard errors. In all corresponding figures, * represents *p* < .05, ** represents *p* < .01, *** represents *p* < .001, and ns represents *p* > .05.
