# Supplementary material for: The mitochondrial unfolded protein response is activated upon hematopoietic stem cell exit from quiescence
Source: Aging Cell. 2018 Mar 24;17(3):e12756. doi: 10.1111/acel.12756 (PMC5946069; doi:10.1111/acel.12756)
Supplement: Supplementary file 1 [file ACEL-17-e12756-s001.docx]

| **FACS Antibodies & Reagents** | **Source** | **Catalog #** | **Clone #** |
| --- | --- | --- | --- |
| CD45.1 PerCP | Biolegend | 110726 | A20 |
| Streptavidin PerCP | Biolegend | 405213 |  |
| Mac1 PerCP | Biolegend | 101230 | M1/70 |
| CD3 Pacific Blue | Biolegend | 100214 | 17A2 |
| Sca1 Pacific Blue | Biolegend | 108120 | D7 |
| Streptavidin APC-Cy7 | Biolegend | 405208 |  |
| c-Kit APC-Cy7 | Biolegend | 105826 | 2B8 |
| CD45.2 Cy7-PE | Biolegend | 109830 | 104 |
| CD150 Cy7-PE | Biolegend | 115914 | TC15-12F12.2 |
| Gr1 Cy7-PE | Biolegend | 108416 | RB6-8C5 |
| c-Kit Cy7-PE | Biolegend | 105813 | 2B8 |
| Streptavidin Cy7-PE | Biolegend | 405206 |  |
| CD3 Biotin | Biolegend | 100304 | 145-2C11 |
| B220 Biotin | Biolegend | 103204 | RA3-6B2 |
| Gr1 Biotin | Biolegend | 108404 | RB6-8C5 |
| CD8a Biotin | Biolegend | 100704 | 53-6.8 |
| Mac1 Biotin | Biolegend | 101204 | M1/70 |
| Ter119 Biotin | Biolegend | 116204 | TER-119 |
| CD4 Biotin | Biolegend | 100404 | GK1.5 |
| CD48 FITC | Biolegend | 103404 | HM48-1 |
| Gr1 FITC | Biolegend | 108406 | RB6-8C5 |
| CD45.2 FITC | eBioscience | 11-0454-85 | 104 |
| Ki67 A488 | Biolegend | 350508 | Ki-67 |
| CD150 PE | Biolegend | 115904 | TC15-12F12.2 |
| CD45.1 PE | Biolegend | 110708 | A20 |
| Mac1 PE | Biolegend | 101208 | M1/70 |
| c-Kit APC | Biolegend | 105812 | 2B8 |
| B220 APC | Biolegend | 103212 | RA3-6B2 |
| Ki67 APC | Biolegend | 350514 | Ki-67 |
| CD48 A647 | Biolegend | 103416 | HM48-1 |
| Fixation buffer | Biolegend | 420801 |  |
| Permeabilization wash buffer | Biolegend | 421002 |  |

Supplementary Table 1: Antibodies used in this study.

| **Gene** | **Primer** | **Sequence** |
| --- | --- | --- |
| ClpP (mouse) | Forward | CTGCCCAATTCCAGAATCAT |
|  | Reverse | TGTAGGCTCTGCTTGGTGTG |
| Hsp10 (mouse) | Forward | CCAAAGGTGGCATTATGCTT |
|  | Reverse | TGACAGGCTCAATCTCTCCA |
| Hsp60 (mouse) | Forward | ACCTGTGACAACCCCTGAAG |
|  | Reverse | TGACACCCTTTCTTCCAACC |
| mtDnaJ (mouse) | Forward | GAGCTGAAGAAGGCATACCG |
|  | Reverse | CAGCTCTCGCTTCTCTGGAT |
| ND4 (mtDNA) (mouse) | Forward | GGAACCAAACTGAACGCCTA |
|  | Reverse | ATGAGGGCAATTAGCAGTGG |
| b2 microglobulin (nDNA) (Mouse) | Forward | TCATTAGGGAGGAGCCAATG |
|  | Reverse | ATCCCCTTTCGTTTTTGCTT |
| Sirt7 (mouse) | Forward | GCACTTGGTTGTCTACACGG |
|  | Reverse | CTTAGGTCGGCAGCACTCAC |
| Cox5a (mouse) | Forward | TTGATGCCTGGGAATTGCGTAAAG |
|  | Reverse | AACAACCTCCAAGATGCGAACAG |
| Cox6a1 (mouse) | Forward | GTTCGTTGCCTACCCTCAC |
|  | Reverse | TCTCTTTACTCATCTTCATAGCCG |
| Pkm2 (mouse) | Forward | TCGCATGCAGCACCTGATT |
|  | Reverse | CCTCGAATAGCTGCAAGTGGTA |
| Hk2 (mouse) | Forward | TGATCGCCTGCTTATTCACGG |
|  | Reverse | AACCGCCTAGAAATCTCCAGA |
| Grp78 (mouse) | Forward | TCATCGGACGCACTTGGAA |
|  | Reverse | CAACCACCTTGAATGGCAAGA |
| PDI (mouse) | Forward | CAAGATCAAGCCCCACCTGAT |
|  | Reverse | AGTTGCCCCAACCAGTACTT |
| Erdj4 (mouse) | Forward | CCCCAGTGTCAAACTGTACCAG |
|  | Reverse | AGCGTTTCCAATTTTCCATAAATT |

Supplementary Table 2: qPCR primers used in this study.
